# Acetylcholine in the gingival epithelium drives the pathogenesis of periodontitis

**DOI:** 10.3389/fcell.2025.1701252

**Published:** 2025-11-20

**Authors:** Shihan Xu, Jiaxin Guo, Shiwen Yang, Bin Cheng, Juan Xia

**Affiliations:** 1 Hospital of Stomatology, Guanghua School of Stomatology, Guangdong Provincial Key Laboratory of Stomatology, Sun Yat-sen University, Guangzhou, China; 2 College of Stomatology, Chongqing Key Laboratory of Oral Diseases and Biomedical Sciences, Chongqing Municipal Key Laboratory of Oral Biomedical Engineering of Higher Education, Chongqing Medical University, Chongqing, China

**Keywords:** single-cell RNA sequencing, spatial transcriptomics, gingival epithelium, periodontitis, acetylcholine, cholinergic signaling

## Abstract

**Background:**

Periodontitis is a highly prevalent chronic inflammatory disease characterized by the progressive destruction of periodontal tissues, which can lead to tooth loss and affect systemic health. This pathological process is driven by both epithelial barrier disruption and a self-perpetuating cycle of dysregulated inflammatory immune responses. Although neurotransmitters, including acetylcholine, are abundant in saliva and gingival crevicular fluid, their role as key mediators of immune homeostasis in the pathogenesis of periodontitis remains poorly understood.

**Methods:**

Utilizing single-cell RNA sequencing (scRNA-seq) data (205,334 cells, 40 human gingival samples) and gingival spatial transcriptomics data (46,230–25 μm^2^ spots), we revealed that the gingival epithelium exhibits the most significant functional reprogramming of neural signaling pathways in the periodontitis state. Through experiments *in vivo* and *in vitro*, we validated the functional role of acetylcholine in periodontitis.

**Results:**

Our findings reveal that cholinergic signals change with the progression of periodontitis and that gingival epithelial cells possess an extensive distribution of non-α7-type nicotinic receptors. The acetylcholine-degrading enzyme, acetylcholinesterase (AChE), is primarily expressed by myeloid immune cells that extensively infiltrate the epithelium, and its expression is significantly upregulated following periodontal treatment. In human oral keratinocytes (HOKs), acetylcholine played a dual role: it promoted epithelial barrier repair by reversing *Porphyromonas gingivalis* (*P. gingivalis*)-induced tight junction disruption, yet it also exacerbated inflammation by upregulating key chemokines and inflammasome components. *In vivo*, mouse models of periodontitis showed that topical application of acetylcholine aggravated periodontal tissue damage.

**Conclusion:**

In conclusion, our results reveal a complex, multifaceted role for acetylcholine in periodontal pathogenesis, highlighting its ability to both protect the epithelial barrier and drive inflammatory tissue destruction. These findings establish a new “neuro-epithelial-immune axis” in the pathogenesis of periodontal disease and reveal potential targets for therapeutic intervention.

## 1 Introduction

Periodontitis is a prevalent chronic inflammatory disease characterized by the progressive destruction of the tooth-supporting apparatus, including the gingiva, periodontal ligament, and alveolar bone (Slots, 2017). Its pathogenesis is driven by a complex interplay between a dysbiotic microbial community and a dysregulated host immune–inflammatory response (Xu et al., 2020; Li et al., 2025b). At the forefront of this host defense is the gingival epithelium (Takahashi et al., 2019; Easter et al., 2024), which acts as a crucial physical barrier and the first line of defense against invading oral pathogens such as *Porphyromonas gingivalis* (*P. gingivalis*) (Wang et al., 2024).

Although the roles of immune cells and microorganisms in periodontitis are well established, the contribution of the nervous system to this intricate disease process remains a frontier of research (Emanuel et al., 2024). The oral mucosa is richly innervated (Moayedi et al., 2022); nerves and neuropeptides influence gingival blood flow and immune cell function (Sakallioglu et al., 2008), thus modulating local inflammation. Acetylcholine is known to regulate inflammation and tissue integrity in various organ systems through the “cholinergic anti-inflammatory pathway,” which typically involves the α7 nicotinic acetylcholine receptor (α7nAChR) and is thought to have a protective effect (Wang et al., 2003; Guzman-Mejia et al., 2018). However, the specific function and regulatory mechanisms of the cholinergic system within the unique inflammatory microenvironment of the gingival tissues are not fully understood.

To comprehensively investigate the cellular and molecular changes underlying periodontitis, we utilized an integrated approach combining single-cell RNA sequencing (scRNA-seq) and our published spatial transcriptomics data (Shen et al., 2024). By analyzing a large cohort of human gingival samples from healthy, periodontitis, and treated periodontitis patients (Caetano et al., 2021; Williams et al., 2021; Chen et al., 2022; Zhu and Huang, 2023), we aimed to determine which neuro-signaling pathways are most affected and map the spatial distribution of their corresponding receptors. In addition, the corresponding functional roles were subsequently validated through *in vivo* and *in vitro* experiments.

In this study, we provide a new paradigm for understanding periodontitis pathogenesis by focusing on the neuro-epithelial axis, which reveals a dual, context-dependent role for acetylcholine and provides a theoretical foundation for developing targeted therapeutic strategies.

## 2 Materials and methods

### 2.1 Cell culture

The immortalized human oral keratinocyte cell line HOK-16B was seeded at a density of 4 × 10^5^ cells/well in six-well plates. Cells were cultured in defined keratinocyte–SFM basal medium (Gibco, NY, United States) supplemented with 0.2% defined keratinocyte–SFM Growth Supplement (Gibco) and 1% penicillin–streptomycin. Cells were maintained at 37 °C in a humidified atmosphere containing 5% CO_2_. Prior to bacterial stimulation, cells were starved for 2 h using growth supplement-free medium.

### 2.2 Bacterial culture

*Porphyromonas gingivalis* (*P. gingivalis,* standard strain ATCC 33277) was cultured anaerobically at 37 °C (80% N_2_, 10% H_2_, and 10% CO_2_) in tryptic soy broth (TSB) supplemented with 0.1% yeast extract, 1 μg/mL menadione, and 5 μg/mL hemin (pH 7.46). Bacteria were inoculated from TSB blood agar plates into liquid medium and grown to the logarithmic phase. Bacterial concentration was measured at 600 nm using spectrophotometry, where OD 1.0 was equivalent to 10^9^/mL of *P. gingivalis*. After low-speed centrifugation, *P. gingivalis* was resuspended in growth medium at a concentration of 10^10^/mL. The multiplicity of infection (MOI) used for bacterial stimulation was 50.

### 2.3 Cell immunofluorescence

HOK-16B cells were seeded at a density of 2 × 10^5^ cells/well in 12-well plates containing coverslips until reaching 50% confluence, followed by stimulation with *P. gingivalis* for 24 h. Coverslips were washed three times with PBS and fixed with 4% paraformaldehyde (PFA) for 20 min at room temperature. Cells were then permeabilized with 0.5% Triton X-100 for 10 min and blocked with blocking buffer (1% BSA/5% normal goat serum) for 1 h at room temperature. Primary antibodies were incubated overnight at 4 °C: mouse anti-NLRP3 (1:500, Proteintech, Wuhan, China, 68102) and rabbit anti-ASC(*PYCARD*) (1:500, Proteintech, 10500). Fluorophore-conjugated secondary antibodies were then applied for 1 h: CoraLite Plus 488-Goat Anti-Mouse Recombinant Secondary Antibody (H + L) (1:2000, Proteintech, RGAM002) and CoraLite Plus 647-Goat Anti-Rabbit Recombinant Secondary Antibody (H + L) (1:2000, Proteintech, RGAR005). CoraLite 594-Phalloidin antibody (1:500, Proteintech, PF00003) was incubated for 20 min at room temperature. Coverslips were mounted using Antifade Mounting Medium with DAPI (Beyotime, Shanghai, China, P0131) and imaged using an Olympus FV 3000 Laser Scanning Confocal Microscope. Pseudocolor adjustments were made for the 594 and 647 channels to enhance visual discrimination.

### 2.4 Enzyme-linked immunosorbent assay

After treating human oral keratinocytes (HOKs) with acetylcholine and *P. gingivalis* either alone or in combination, the cells were cultured in new medium (for the removal of *P. gingivalis*) for 2 h, and the supernatant was collected. The levels of CXCL1 and CXCL8 were then measured using an enzyme-linked immunosorbent assay (ELISA) kit (Proteintech, KE00133 and KE00453), according to the manufacturer’s instructions. In brief, samples and standards were added to pre-coated wells, followed by incubation with detection antibodies and HRP-conjugated secondary antibodies. The enzymatic reaction was initiated by adding a TMB substrate and stopped with an acidic solution. The optical density was read at 450 nm using a microplate reader, and protein concentrations were calculated based on a standard curve.

### 2.5 RNA isolation and quantitative real-time PCR

Total RNA was isolated using TRIzol reagent (Invitrogen, United States) and reverse-transcribed to first-strand cDNA using the PrimeScript RT Reagent Kit with gDNA Eraser (Vazyme Biotech, China). Quantitative real-time PCR (qPCR) was performed using SYBR qPCR Master Mix (Yeasen Biotechnology, China) on a Roche LightCycler 480 II System. Human-specific primers were synthesized by Generay Biotech: *OCLN* (forward 5′-GACTTCAGGCAGCCTCGTTAC-3′; reverse 5′-GCCAGTTGTGTAGTCTGTCTCA-3′), *CLDN1* (forward 5′-CCTCCTGGGAGTGATAGCAAT-3′; reverse 5′-GGCAACTAAAATAGCCAGACCT-3′), *CDH1* (forward 5′-CGAGAGCTACACGTTCACGG-3′; reverse 5′-GGGTGTCGAGGGAAAAATAGG-3′), and *GAPDH*: (forward 5′-GGAGCGAGATCCCTCCAAAAT-3′; reverse 5′GGCTGTTGTCATACTTCTCATGG-3′). Gene expression levels were calculated using the 2^−ΔΔCT^ method and normalized to GAPDH as the internal reference gene.

### 2.6 Patient recruitment and tissue sample collection

Gingival tissue samples from three periodontitis patients were collected from patients undergoing tooth extraction at the Department of Oral and Maxillofacial Surgery, Affiliated Stomatology Hospital of Sun Yat-sen University (Guangzhou, China). All sample collection procedures complied with China’s “Good Clinical Practice for Drug Clinical Trials,” ICH-GCP guidelines, and relevant regulations and were approved by the Medical Ethics Committee of the Affiliated Stomatology Hospital of Sun Yat-sen University (Approval No.: KQEC-2022-14-01). Informed consent was obtained from all participants prior to enrollment. The inclusion criteria for periodontitis patients included the affected tooth having a probing depth (PD) of > 4 mm and a bleeding index (BI) of ≥ 3 upon probing. Exclusion criteria included patients with concurrent systemic diseases.

### 2.7 Tissue processing and immunofluorescence

Collected gingival tissues were placed in Hank’s balanced salt solution and fixed in 4% PFA for 10 min, followed by further fixation in 4% PFA at 4 °C for 24 h. Tissues were then dehydrated through a graded ethanol series (70%–100%), cleared in xylene, and embedded in paraffin. Continuous 5 μm sections were prepared, baked at 60 °C for 2 h, dewaxed in xylene, and rehydrated through a graded ethanol series for immunofluorescence staining. Antigen retrieval was performed using citrate buffer (pH 6.0) at 95 °C, followed by blocking with blocking buffer (1% BSA/5% normal goat serum) for 1 h at room temperature. Sections were incubated overnight at 4 °C with rabbit anti-acetylcholinesterase polyclonal antibody (1:500, Proteintech, 17975-1-AP), followed by incubation with CoraLite 488-conjugated Goat Anti-Rabbit IgG (H + L) (1:2000, Proteintech, SA00013-2) for 1 h. Sections were mounted using Antifade Mounting Medium with DAPI (Beyotime, P0131) and imaged using an Olympus FV 3000 Laser Scanning Confocal Microscope.

### 2.8 Mouse experimental periodontitis model

The Institutional Animal Care and Use Committee (IACUC) at Sun Yat-sen University reviewed and approved the animal use protocol (SYSU-IACUC-2024-000348). This evaluation adhered to the standards of the Animal Welfare Act and the U.S. Public Health Service’s “Guide for the Care and Use of Laboratory Animals.” All animals were housed in a specific pathogen-free (SPF) environment with controlled humidity and temperature on a 12-h light–dark cycle.

Mice were randomly assigned to the HC (healthy control), PD (periodontitis), and PD + ACh treatment groups. Under anesthesia (ketamine/xylazine), periodontitis was induced by placing a 0.15-mm-diameter silk ligature around the maxillary second molars, ligated at both the mesial and distal sides. The ligature was tied securely on the buccal side, and a 1 × 10^10^ CFU/mL *P. gingivalis* (Pg) suspension was applied every 3 days under isoflurane anesthesia. Freshly prepared acetylcholine solution was administered daily in the PD + ACh treatment group, while the PD group received a control aqueous solution of the same pH value. After 14 days, the mice were euthanized, and maxillary blocks were harvested and fixed in 4% paraformaldehyde. Mice that experienced premature ligature loss were excluded. Bone loss was assessed using micro-computed tomography (micro-CT). Additionally, a random data-processing method was used to mitigate potential confounding factors.

After decalcification with disodium ethylenediaminetetraacetate (EDTA), the mouse maxillae were embedded in paraffin, sectioned into 5 µm-thick slices, and stained with hematoxylin and eosin (H&E). Serial sections of the buccal and lingual sides of the periodontal area were observed under a microscope, and images were captured using a PANNORAMIC SCAN scanner.

### 2.9 Statistical analysis

Data were analyzed using GraphPad Prism 10 and expressed as the mean ± standard error of the mean (SEM). Statistical significance was evaluated using t-tests (for two-group comparisons) and one-way ANOVA (three or more groups, followed by Tukey’s HSD post hoc test). Significant differences were denoted as follows: *p < 0.05, **p < 0.01, and ***p < 0.001.

### 2.10 RNA sequencing

Library preparation: Total RNA was isolated from HOKs using TRIzol reagent. RNA integrity and concentration were evaluated using an Agilent 2100 Bioanalyzer. Polyadenylated mRNA was enriched via oligo(dT)-conjugated magnetic beads and subsequently fragmented in NEB fragmentation buffer containing divalent cations. First-strand cDNA synthesis was performed using the M-MuLV reverse transcriptase system, followed by second-strand synthesis using DNA polymerase I and deoxynucleotide triphosphates (dNTPs). The resulting double-stranded cDNA was purified and subjected to end repair, A-tailing, and adapter ligation. cDNA fragments of approximately 250–300 bp were size-selected using AMPure XP beads, PCR-amplified, and further purified with AMPure XP beads to generate the final library. Initial quantification was conducted using a Qubit 2.0 Fluorometer, followed by dilution of libraries to 1.5 ng/μL. Insert size distribution was assessed using an Agilent 2100 Bioanalyzer, and accurate quantification of library effective concentration (>1.5 nM) was achieved via qRT-PCR to ensure high-quality preparation.

Sequencing and quality control: Libraries were pooled according to effective concentration and subjected to Illumina sequencing, generating 150 bp paired-end reads. During sequencing, four fluorescence-labeled dNTPs, DNA polymerase, and adapter primers were added to the flow cell for amplification. The optical signals were converted to sequencing peaks using computer software to obtain sequence information. Raw image data were base-called using CASAVA into sequence data (reads) in FASTQ format, followed by filtering to remove low-quality data.

Gene expression quantification: Reference genome indices were built using HISAT2 (v2.0.5), and paired-end clean reads were aligned to the human reference genome using HISAT2 (v2.0.5). Read counts mapped to each gene were calculated using featureCounts (v1.5.0-p3).

Data analysis: Differential expression analysis between groups was performed using DESeq2 (v1.46.0). Gene set enrichment analysis (GSEA) for GO functional annotation was conducted using clusterProfiler (v4.14.4). GSEA was performed using fgsea (v1.32.2) and msigdb (v1.14.0).

### 2.11 Single-cell data analysis

Data acquisition: Previously published human gingival single-cell datasets, including GSE152042, GSE164241, GSE171213, and GSE207502, were integrated. These datasets were carefully selected to represent both healthy and diseased states, encompassing healthy, periodontitis, and treated periodontitis samples, while excluding non-gingival periodontal tissues. The final integrated dataset comprised 40 samples and 205,334 cells.

Integration and clustering: All analyses were performed using the Seurat (v5.1.0) and Harmony (v1.2.3) R packages. Following quality control, 152,073 cells were retained for downstream analysis. After log normalization, highly variable genes were identified, and principal component analysis (PCA) was performed. Batch effects were corrected using Harmony. Cell clusters were identified using Seurat’s Louvain algorithm and visualized with UMAP dimensionality reduction. Cell types were annotated based on canonical markers, and immune cell subtypes were further refined through secondary clustering.

Data analysis: Neural receptor pathway-related gene sets (GO:0099637, GO:0099601, GO:0098962, and GO:0099529; R-HSA-112314) were obtained from the MsigDB website and intersected with highly variable genes (HVGs) from different cell clusters. These were further intersected with significantly differentially expressed genes (DEGs) in epithelial cell populations across groups (healthy and periodontitis), followed by KEGG pathway clustering using clusterProfiler (v4.14.4).

### 2.12 Spatial transcriptomics data analysis

Stereo-sequencing data were processed as previously described (Shen et al., 2024). Data were captured on a silicon chip covered with 220 nm × 220 nm DNA nanoballs (DNBs). The raw spatial expression matrix was converted into 25 μm^2^ pseudo-spots (50 × 50 DNBs per spot) and imported into a Seurat object, where it was normalized using the SCTransform function.

Subsequently, we performed PCA for dimensionality reduction. A K-nearest neighbor (KNN) graph was constructed based on the Euclidean distance, and cell clusters were identified using the FindClusters function. The FindAllMarkers function was then used to identify marker genes for each cluster, allowing us to classify the four cell types based on these markers. The gingival spots from Stereo-seq were color-coded to reflect the expression levels of key genes, including cell type identification markers (*KRT15*, *COL1A1*, *PECAM1*, and *PTPRC*) and acetylcholine receptors.

## 3 Results

### 3.1 Single-cell and spatial transcriptomic atlases reveal the cellular landscape of gingival tissues in periodontal health and disease

**FIGURE 1:**
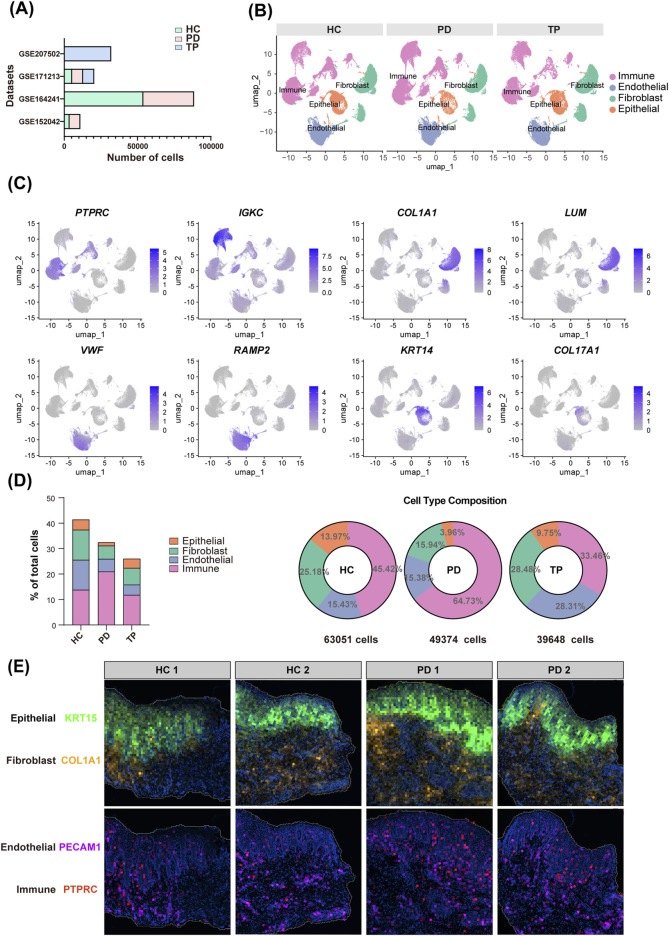
Gingival cellular dynamics landscape during periodontitis progression. **(A)** Dataset sources (y-axis) and sequenced cell count (x-axis) of gingival single-cell RNA sequencing (scRNA-seq). Groups: healthy control (HC, blue), periodontitis (PD, red), and treated periodontitis (TP, green). **(B)** Uniform manifold approximation and projection (UMAP) visualization of the integrated single-cell data from HC, PD, and TP groups. **(C)** Marker gene validation: representative transcripts defining the major cell lineages. **(D)** The total cell percentage for different cell types across the entire dataset (left) and the proportional composition of cell types within each group (right). **(E)** Spatial mapping of the gene expressions of KRT15, COL1A1, PECAM1, and PTPRC. Groups: healthy controls (HC) and periodontitis (PD).

The scRNA-seq datasets comprised 205,334 cells from 40 samples across three clinical categories: 19 healthy controls (HC), 15 periodontitis patients (PD), and 6 treated periodontitis cases (TP) (Table 1; Figure 1A) (Caetano et al., 2021; Williams et al., 2021; Chen et al., 2022; Zhu and Huang, 2023). The spatial transcriptomics data included HC and PD samples with 46,230 spots at a resolution of 25 μm^2^ (Shen et al., 2024).

**TABLE 1:** Sources of single-cell sequencing datasets.

GSE ID	Healthy control (HC)	Periodontitis (PD)	Treated periodontitis (TP)	PMID	Publication date
152042	2	2	0	33393902	January 2021
164241	13	8	0	34129837	July 2021
171213	4	5	3	35154475	January 2022
207502	0	0	3	36947913	June 2023

Gingival single-cell RNA sequencing (scRNA-seq) datasets, with disease states explicitly annotated in the original publications. HC, healthy controls; PD, periodontitis; TP, treated periodontitis.

After quality control, we classified the single-cell populations using classical markers (Williams et al., 2021): immune cells (*PTPRC* and *IGKC*), fibroblasts (*COL1A1* and *LUM*), endothelial cells (*VWF* and *RAMP2*), and epithelial cells (*KRT14* and *COL17A1*) (Figures 1B,C). Quantitative cell analysis identified epithelial cells as the least abundant population, while immune cells were the most predominant. The PD group showed a pronounced infiltration of immune cells and a concomitant reduction in epithelial cells (Figure 1D), which reflects the pathological inflammatory tissue destruction observed in periodontitis (Kim et al., 2024).

Spatial transcriptomic analysis further revealed cellular distributions within the gingiva (Figure 1E). The expression of *KRT15*, a basal epithelial marker, clearly delineated the boundary between the epithelium and the lamina propria, while *PTPRC*, a pan-immune marker, revealed the spatial infiltration of immune cells in periodontitis.

### 3.2 Epithelial cells show the most pronounced neurotransmitter signaling-related gene reprogramming during periodontitis

**FIGURE 2:**
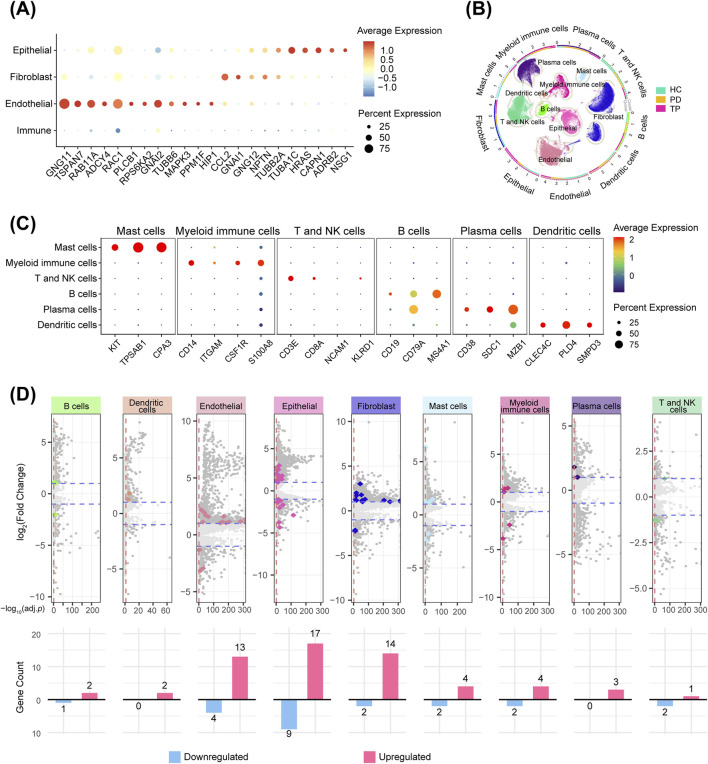
Gingival neurotransmitter signaling-related gene changes during periodontitis progression. **(A)** Cross-analysis of highly variable genes (HVGs, top 2000) and neurotransmitter signaling-related genes (Table 2) in four gingival cell types. **(B)** Circular UMAP plots of cell subpopulations. The outer ring and scatter plot colors indicate different cell types, while the inner ring colors represent the cell number ratio among the groups. **(C)** Markers used for immune cell identification, visualized with a bubble plot. **(D)** Volcano plots showing DEGs between the PD and HC groups for each cell type. Darker dots represent genes with a |log2-fold change| > 1 and an adjusted *p*-value <0.05. Colored dots represent genes from the neurotransmitter signaling-related gene sets, and the bar graphs show their counts (upregulated and downregulated in periodontitis).

A cross-analysis between HVGs and neurotransmitter signaling-related gene sets (Table 2) indicated that endothelial cells exhibited the most extensive potential for neural interactions (Figure 2A). Endothelial cells highly express G proteins (*GNG11* and *GNAI2*), which act as “switches” for neurotransmitter receptor (e.g., GPCR) signal transduction (Liccardo et al., 2022), along with key regulators of signal transduction and vesicle transport, such as *RAC1* and *RAB11A* (Hulsbusch et al., 2015). This observation can be attributed to the dense sympathetic and sensory innervation of microvessels, where endothelial cells rapidly transmit neural signals to regulate local blood flow and inflammatory extravasation (de Juan et al., 2019; Forrester et al., 2024). Epithelial cells characteristically express *ADRB2* (β-2 adrenergic receptor)—a classic neurotransmitter receptor (Li et al., 2025a)—and *NSG1*, which can participate in neurotransmitter receptor endocytosis and transport (Barford et al., 2017), demonstrating the potential of epithelial cells to receive neurotransmitter signals. Fibroblasts express *GNAI1* (G protein subunit) and *NPTN* (neuroplastin, an adhesion molecule) (Ilic et al., 2021), suggesting that fibroblasts might also respond to neural signals. In contrast, immune cells showed the minimal signatures of direct neural interactions, suggesting that their regulation primarily occurs through indirect neuro-stromal-immune crosstalk (Hodo et al., 2020).

**TABLE 2:** Neurotransmitter signaling-related gene sets from the Molecular Signatures Database (MSigDB).

Source	Name	Description
GO 0099637	GOBP_NEUROTRANSMITTER_RECEPTOR_TRANSPORT	The directed movement of neurotransmitter receptors
GO 0099601	GOBP_REGULATION_OF_NEUROTRANSMITTER_RECEPTOR_ACTIVITY	Any process that modulates the frequency, rate, or extent of neurotransmitter receptor activity. Modulation may be via an effect on ligand affinity or effector function such as ion selectivity or pore opening/closing in ionotropic receptors
GO 0098962	GOBP_REGULATION_OF_POSTSYNAPTIC_NEUROTRANSMITTER_RECEPTOR_ACTIVITY	Any process that modulates the frequency, rate, or extent of neurotransmitter receptor activity involved in synaptic transmission. Modulation may be via an effect on ligand affinity or effector function such as ion selectivity or pore opening/closing in ionotropic receptors
GO 0099529	GOMF_NEUROTRANSMITTER_RECEPTOR_ACTIVITY_INVOLVED_IN_REGULATION_OF_POSTSYNAPTIC_MEMBRANE_POTENTIAL	Neurotransmitter receptor activity occurring in the postsynaptic membrane that is involved in regulating postsynaptic membrane potential, either directly (ionotropic receptors) or indirectly (e.g., via GPCR activation of an ion channel)
R-HSA-112314	REACTOME_NEUROTRANSMITTER_RECEPTORS_AND_POSTSYNAPTIC_SIGNAL_TRANSMISSION	Neurotransmitter receptors and postsynaptic signal transmission

Immunophenotyping using canonical markers identified distinct immune cell subpopulations (Figures 2B,C), enabling a comparative analysis between the periodontitis and healthy groups across all signature subpopulations. The cross-analysis of significant DEGs (*p* < 0.05), with neurotransmitter signaling-related gene sets, revealed that, among all cell types, gingival epithelial cells exhibited the most distinct rearrangement of neurotransmitter signaling-related genes during the progression of periodontitis (Figure 2D). This finding is particularly significant as the epithelium acts as the primary barrier against periodontal pathogens (Chang et al., 2021). Its unique neurotransmitter-mediated reactivity prompted our further investigation into its potential regulatory mechanisms.

### 3.3 Acetylcholine signaling and receptors in the periodontal epithelium

**FIGURE 3:**
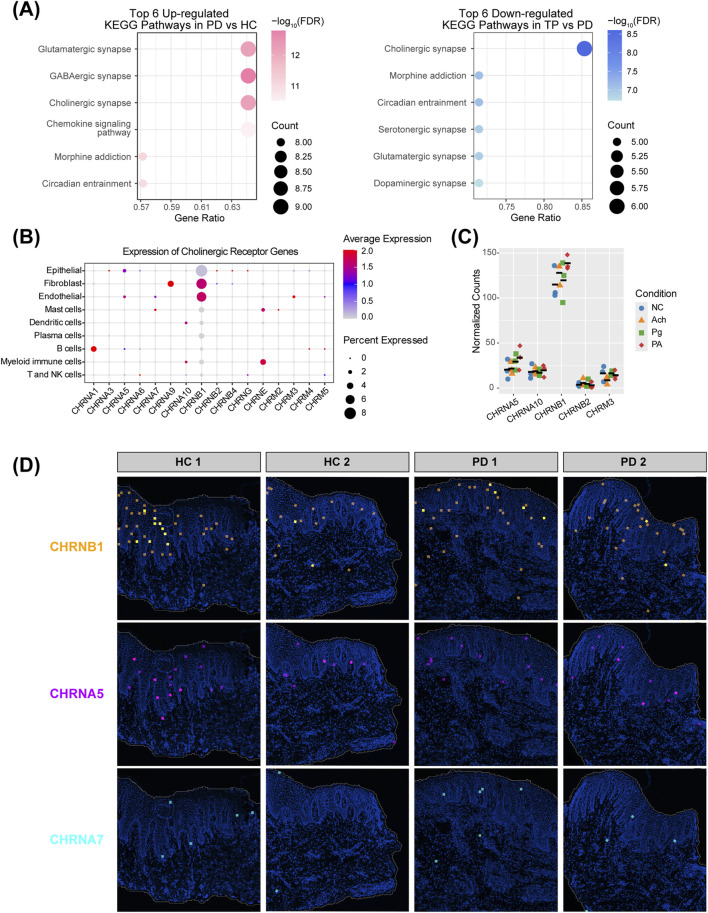
Acetylcholine signaling and receptor distribution in the periodontal epithelium. **(A)** Kyoto Encyclopedia of Genes and Genomes (KEGG) enrichment of epithelial neurotransmitter signaling-related DEGs. Left: upregulated pathways in PD vs. HC; right: downregulated pathways in TP vs. PD. **(B)** Expression profiles of acetylcholine receptors across different cell types. Receptors not detected in any cells were excluded. **(C)** Transcript counts of acetylcholine receptor genes from HOKs RNA-seq data. Five receptors detected in all 12 samples (n = 3 per group), with colored shapes representing groups and black bars indicating the mean. Receptors with low expression (zero counts in some samples) were excluded. **(D)** Expression of acetylcholine receptors *CHRNB1*, *CHRNA5*, and *CHRNA7* in gingival spatial transcriptomics data.

Next, we performed enrichment analysis on epithelial DEGs related to neurotransmitter signaling under various periodontitis conditions. Kyoto Encyclopedia of Genes and Genomes (KEGG) analysis of upregulated genes in PD vs. HC showed that glutamatergic synapse, GABAergic synapse, cholinergic synapse, and chemokine signaling pathways were the most significantly enriched. Among these, the cholinergic synapse pathway showed the most pronounced downregulation in the comparison between TP and PD (Figure 3A), suggesting its dynamic regulation during disease remission. The evolutionarily conserved cholinergic system mediates neuron–peripheral organ communication (Alen, 2022), as exemplified by a 14-fold increase in acetylcholine observed in atopic dermatitis (Wessler et al., 2003). Previous studies have shown that gingival tissues express characteristic acetylcholine receptors (nicotinic/muscarinic) and metabolic enzymes, including acetylcholinesterase (AChE) (Arredondo et al., 2003; Meric et al., 2019). More importantly, acetylcholine levels are significantly elevated in the saliva and gingival crevicular fluid of periodontitis patients (Apatzidou et al., 2018), suggesting that cholinergic signaling may contribute to the progression of periodontitis, although its specific role remains unclear.

Fibroblasts and endothelial cells show a high expression of the cholinergic receptor nicotinic beta 1 subunit (*CHRNB1*), whereas epithelial cells exhibit a broader but lower level. Given that the epithelium acts as the first barrier against bacterial invasion in periodontitis, is in direct contact with acetylcholine in the gingival crevicular fluid and saliva, and exhibits dynamic changes in the cholinergic synapse pathway across different stages of periodontitis (Figure 3A), we focused our subsequent investigation on epithelial cells. In addition to *CHRNB1*, epithelial cells also show expression of the cholinergic receptor nicotinic alpha 5 subunit (*CHRNA5*), with negligible expression of *CHRNA7*, which is considered an anti-inflammatory “pocket” (Figure 3B) (Wang et al., 2003; Guzman-Mejia et al., 2018). We performed RNA-seq on HOKs, and the captured transcripts showed a similar proportion, with *CHRNB1* far exceeding other receptors, followed by *CHRNA5*, and very low expression of *CHRNA7*. This expression pattern was further corroborated by the spatial transcriptomics landscape (Figures 3C,D). This unique receptor profile suggests that the role of acetylcholine in the gingival epithelium may differ from the classic α7nAChR-dependent anti-inflammatory pathway.

### 3.4 Expression patterns of gingival cholinergic metabolic enzymes

The core acetylcholine synthesis gene *CHAT* was not detected in the whole gingiva scRNA data (data not shown) (Ferreira-Vieira et al., 2016), indicating that resident gingival cells do not produce acetylcholine themselves. Acetylcholine in gingival crevicular fluid and saliva is, therefore, likely to originate mainly from neuronal release. Interestingly, *AChE* was consistently detected, suggesting active regulation of cholinergic signaling in the gingiva (Korabecny and Soukup, 2021).

**FIGURE 4:**
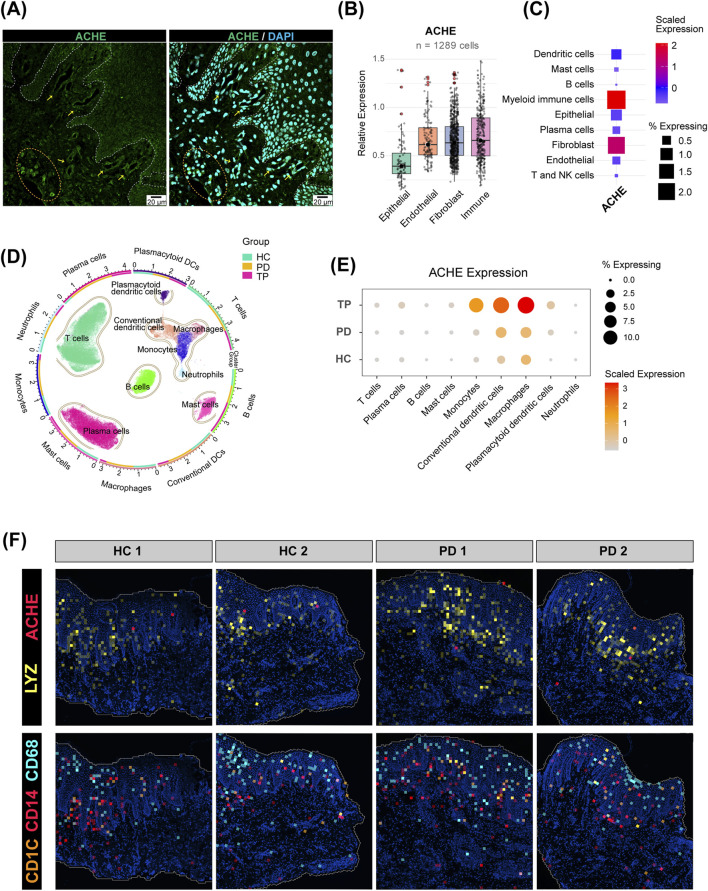
Expression pattern of acetylcholinesterase (AChE) in the gingiva. **(A)** Immunofluorescence staining of the human gingival epithelium. Cell nuclei are stained with DAPI (blue), and AChE is labeled in green (scale bar: 20 µm). The dashed line delineates the epithelial–connective tissue boundary. **(B)** Relative expression of AChE in the four major cell types from the scRNA-seq data. Only cells expressing AChE (expression >0) were included (n = 1,289). The center line indicates the median. **(C)** Expression of AChE in further refined cell subpopulations. **(D)** UMAP plot of isolated immune cell subpopulations. The outer ring and scatter plot colors indicate different cell types, while the inner ring colors represent the cell number ratio among the groups. **(E)** Expression of AChE in immune cells from the HC, PD, and TP groups. **(F)** Spatial distribution of AChE and myeloid immune cell marker genes in the gingiva.

Immunofluorescence localized the ACHE protein primarily to immune clusters and vascular endothelium (Figure 4A, circles and arrows). The boxplot analysis shows that the median *ACHE* expression level in epithelial cells was lower than that in other cell types (Figure 4B). Overall, myeloid immune cells expressed the most *ACHE* (Figure 4C), even more than endothelial cells and fibroblasts. Further subdivision of immune cells revealed that macrophages, conventional dendritic cells, and monocytes highly expressed *ACHE* after periodontal treatment (Figures 4D,E). Corroborating this, spatial transcriptomics maps showed the distribution of these cell types with marker genes (Mulder et al., 2021): *LYZ* (myeloid immune cells), *CD68* (macrophages), *CD14* (monocytes), and *CD1C* (dendritic cells) (Figure 4F), revealing that a large number of myeloid immune cells were observed infiltrating the epithelial area. As AChE is a typical secretory hydrolase (Walczak-Nowicka and Herbet, 2021), the significant upregulation of *ACHE* by myeloid immune cells after periodontal treatment could facilitate the normalization of epithelial cholinergic signaling.

### 3.5 Acetylcholine promotes periodontal epithelial barrier repair and chemokine expression

**FIGURE 5:**
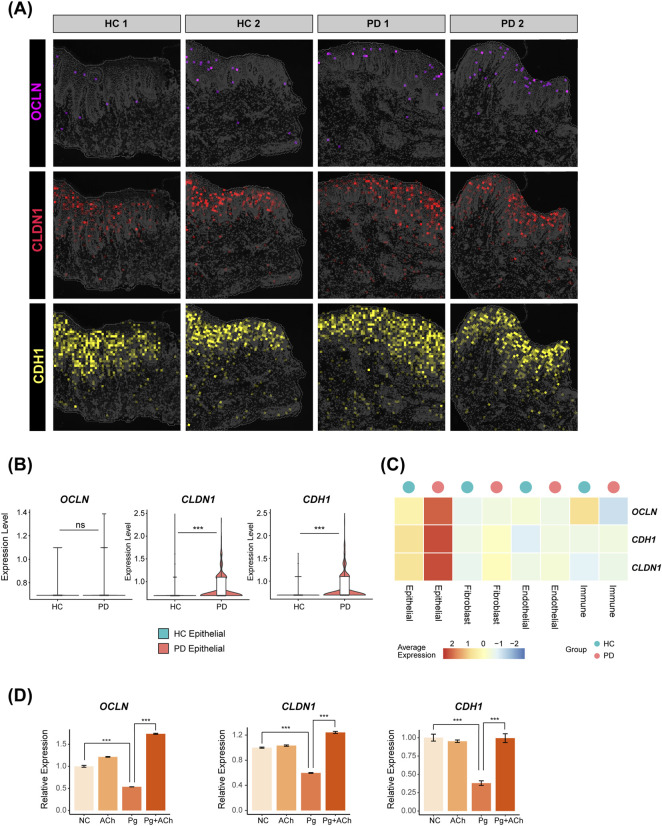
Distribution of tight junction genes in the periodontal gingival epithelium and their regulation by acetylcholine. **(A)** Expressions of OCLN, CLDN1, and CDH1 in spatial transcriptomics data. **(B)** Differential comparison of OCLN, CLDN1, and CDH1 in the epithelial subpopulation of HC versus PD groups (***, *p* < 0.001). **(C)** Heatmap showing the expressions of OCLN, CLDN1, and CDH1 for each cluster in HC and PD gingiva. **(D)** Quantitative polymerase chain reaction (qPCR) validation in HOKs. Data are presented as the mean ± standard error of the mean (SEM). ***, *p* < 0.001.

The periodontitis-affected gingival epithelium highly expressed tight junction genes claudin-1 (*CLDN1*) and E-cadherin (*CDH1*) (*p* < 0.001), while the average expression of occludin (*OCLN*) was also elevated, although this was not statistically significant (Figures 5A–C) (Fernandez-Lainez et al., 2023; Wen et al., 2024). Infection of HOKs with *P. gingivalis*, the key periodontal pathogen (Lamont et al., 2022), significantly downregulated *OCLN*, *CLDN1*, and *CDH1*. However, pretreatment with acetylcholine significantly reversed this epithelial barrier disruption (Figure 5D), indicating that acetylcholine may restore epithelial barrier function by maintaining tight junction integrity during *P. gingivalis* attack.

**FIGURE 6:**
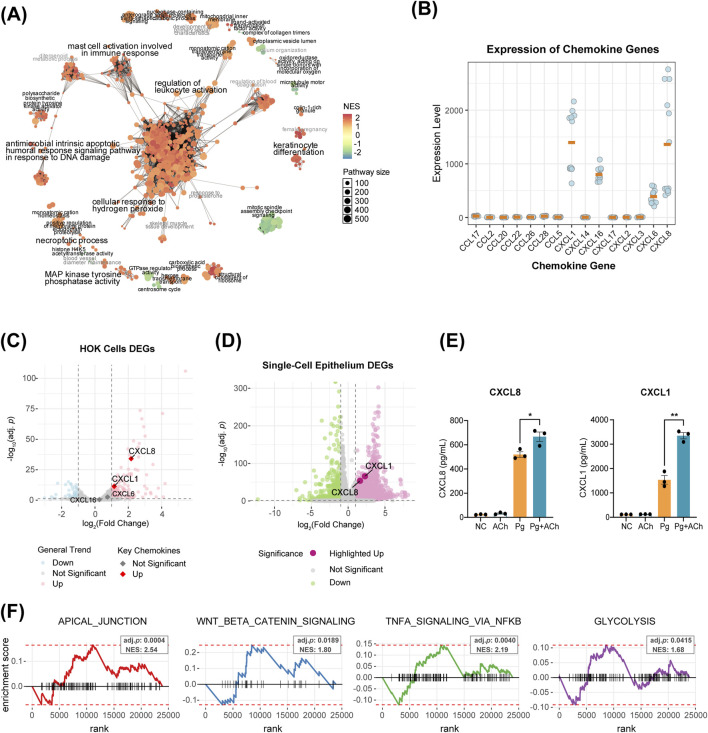
Acetylcholine orchestrates a multi-dimensional defense program in epithelial cells. **(A)** Network of GSEA-GO enrichment analysis for Pg vs. NC DEGs. Each node represents a GO term, with circle size and color indicating the pathway size and the NES score, respectively. Node-sharing clusters represent functionally related term groups. **(B)** Transcript counts of CXCL and CCL family genes in HOKs RNA-seq data. Only genes with non-zero counts across all 12 samples are displayed, with orange bars indicating the mean. **(C)** Volcano plot of DEGs in Pg vs. NC comparison. Colored dots represent significantly differential expression (|log2 fold change| > 1, adjusted *p*-value <0.05). **(D)** Volcano plot of PD vs. HC DEGs in epithelial cells from single-cell data. Colored dots represent significantly differential expression (|log2 fold change| > 1, adjusted *p*-value <0.05). **(E)** Enzyme-linked immunosorbent assay (ELISA) results of HOK supernatants for CXCL1 and CXCL8. *, *p* < 0.05 and **, *p* < 0.01. **(F)** Enrichment analysis of DEGs from the Pg + Ach vs. Pg groups using the fgsea function, showing four significantly enriched functional categories [adjusted *p* < 0.05, normalized enrichment score (NES) > 1.5].

To further investigate the effects of acetylcholine on the epithelium under periodontitis environment, we performed RNA-seq analysis on HOKs under four experimental conditions: untreated control (NC), acetylcholine (ACh, 100 nM), *P. gingivalis* infection (Pg, MOI = 50), and combined *P. gingivalis* infection with acetylcholine treatment (Pg + ACh). Gene set enrichment analysis of Gene Ontology (GSEA-GO) revealed that the *P. gingivalis* attack induced the upregulation of several biological processes, such as immune regulation (leukocyte/mast cell activation), hydrogen peroxide response, and antibacterial apoptosis triggered by DNA damage (Figure 6A).

Transcriptome analysis showed that the main chemokines expressed by HOKs were *CXCL1*, *CXCL6*, *CXCL8*, and *CXCL16* (Figure 6B). Of these, *CXCL1* and *CXCL8* were significantly upregulated after *P. gingivalis* infection (Figure 6C) and also upregulated in periodontitis epithelial cells from scRNA-seq data (Figure 6D). ELISA of the supernatant after infection or combined treatment showed that Pg infection promoted the secretion of CXCL1 and CXCL8 proteins by HOKs, and acetylcholine exacerbated this process, further enhancing CXCL1 and CXCL8 secretions (Figure 5E).

Enrichment analysis of DEGs between the Pg + ACh and Pg groups further revealed important regulatory patterns of acetylcholine on the infected epithelium (Figure 6F): upregulation of epithelial repair pathways (APICAL_JUNCTION and WNT/BETA_CATENIN signaling), which corroborates the previous barrier protection findings (Figure 5D) (Mak et al., 2013; Kosumi et al., 2022); and upregulation of TNFA SIGNALING VIA NFKB and GLYCOLYSIS, indicating that acetylcholine exacerbates the inflammatory response and promotes reprogramming of cellular energy metabolism (Chen et al., 2023). In summary, acetylcholine exhibits a dual role in the context of *P. gingivalis* infection: on one hand, it enhances chemokine-mediated immune recruitment [potentially exacerbating inflammatory damage (Barreiro et al., 2010)], while on the other hand, it promotes epithelial barrier repair and cellular energy supply, demonstrating its complex regulatory function in host–pathogen interactions.

### 3.6 Acetylcholine exacerbates inflammasome production and periodontal destruction

**FIGURE 7:**
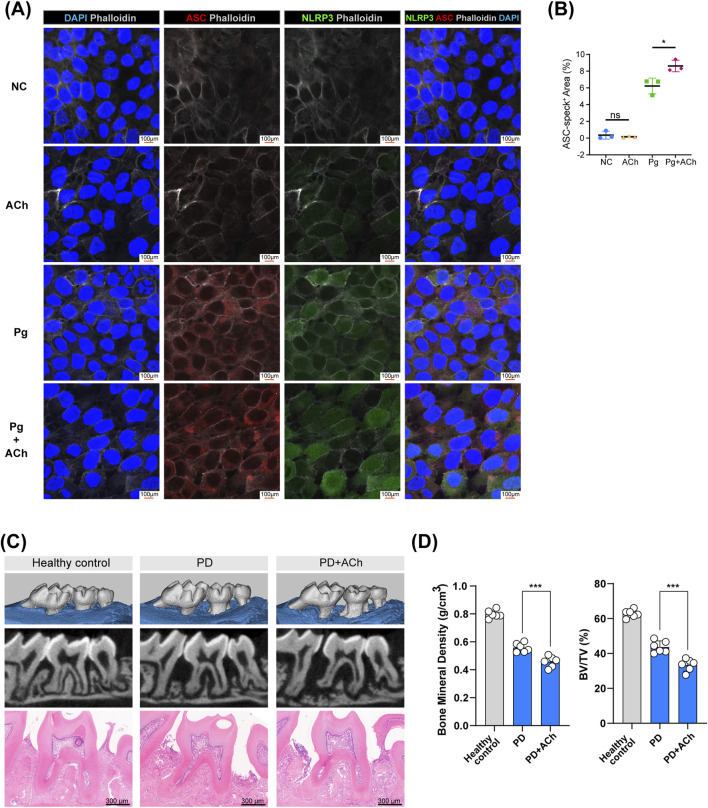
Acetylcholine promotes inflammasome activation and bone loss in periodontitis. **(A)** Representative confocal images of HOK inflammasome activation after treating with P. gingivalis (Pg), acetylcholine (ACh), or both for 24 h. ASC (red), NLRP3 (green), phalloidin (white), and DAPI (blue); scale bar: 100 µm. **(B)** Quantification of ASC-positive areas. *, *p* < 0.05; ns, no difference. **(C)** Micro-CT scans and H&E staining of maxillary bones from each group (control, PD, and PD + ACh). Scale bar: 300 µm. (n = 6/group) **(D)** Analysis of bone mineral density (BMD) and the bone volume/tissue volume (BV/TV) ratio in the second molar area from micro-CT scans, represented by individual data points (n = 6/group). ***, *p* < 0.001.

HOKs were treated with acetylcholine alone, *P. gingivalis* alone, or *P. gingivalis* with acetylcholine for 24 h. Acetylcholine enhanced the expression of inflammasome components, including NOD-like receptor family pyrin domain-containing 3 (NLRP3) and apoptosis-associated speck-like protein containing a CARD (ASC, also called *PYCARD*) (Figures 7A,B). Similar to chemokines, while inflammasome activation helps clear pathogens, it can exacerbate tissue damage (Sharma and Kanneganti, 2021; Fu and Wu, 2023).

Mouse experimental periodontitis models, established through silk ligation and *P. gingivalis* inoculation for 14 days, demonstrated that daily application of acetylcholine exacerbated periodontal tissue destruction (Figure 7C), leading to lower alveolar bone density (Figure 7D). Acetylcholine’s dual effects—barrier protection and pro-inflammatory enhancement—ultimately worsen tissue destruction in the periodontitis environment.

## 4 Discussion

In this study, scRNA-seq and spatial transcriptomics analyses, combined with *in vivo* and *in vitro* experiments, reveal a novel dual role for acetylcholine signaling in periodontitis. We first identified that although multiple cell types interact with neural signals, gingival epithelial cells undergo the most significant reprogramming of neurotransmitter-related gene expression, particularly involving the cholinergic synapse pathway, during the progression from health to periodontitis. Investigating this further, we found that epithelial cells express a distinct cholinergic receptor profile, characterized by high levels of non-α7 nicotinic receptors *CHRNB1* and *CHRNA5*, with minimal expression of the canonical anti-inflammatory receptor *CHRNA7*. Regarding the source and regulation of acetylcholine, we observed negligible expression of the synthesis enzyme CHAT in resident gingival cells, suggesting that acetylcholine likely originates from neural release or fluids such as saliva. Conversely, the degrading enzyme AChE is prominently expressed, primarily by infiltrating myeloid immune cells, and its levels increase significantly following periodontal treatment, indicating active regulation of local acetylcholine concentrations. Functionally, *in vitro* experiments on HOKs demonstrated a dual role for acetylcholine in the context of *P. gingivalis* infection: it promoted epithelial barrier repair by counteracting pathogen-induced tight junction disruption and simultaneously exacerbated the inflammatory response by increasing the expression and secretion of pro-inflammatory chemokines (CXCL1 and CXCL8) and inflammasome components (NLRP3 and ASC). This observation is particularly significant, given that epithelial cells serve as the first line of defense against periodontal pathogenic bacteria and are in direct contact with a large amount of neurotransmitters in saliva and gingival crevicular fluid (Sakallioglu et al., 2008; Takahashi et al., 2019). Crucially, *in vivo* validation in a mouse model showed that the net effect of topical acetylcholine application was an aggravation of periodontal tissue destruction and bone loss. Collectively, these findings establish a previously unappreciated “neuro-epithelial-immune axis” driven by acetylcholine in periodontitis.

A key finding of our study is the expression profile of acetylcholine receptors in gingival epithelial cells, with *CHRNB1* and *CHRNA5* being highly expressed, while the classic anti-inflammatory receptor *CHRNA7* shows minimal expression (Burke et al., 2024). This pattern, also confirmed in HOKs, strongly suggests that acetylcholine does not exert its classic α7nAChR-mediated anti-inflammatory effects in the gingival epithelium (Hone and McIntosh, 2023).

Furthermore, our analysis of cholinergic metabolic enzymes provides an insight into the gingival acetylcholine microenvironment. The absence of *CHAT* transcription in resident cells suggests that acetylcholine may originate from the dense local neural network (Ferreira-Vieira et al., 2016) and from saliva and gingival crevicular fluid. In contrast, the prominent expression of *AChE* in myeloid immune cells and the vascular endothelium highlights the active capacity of the gingiva to regulate acetylcholine levels (Petsakou et al., 2023). The spatial transcriptomics landscape demonstrates a substantial infiltration of myeloid immune cells into the periodontal epithelium, and the significant upregulation of *AChE* in these myeloid cells after periodontal treatment suggests a potential feedback loop that modulates the local acetylcholine concentration, thereby facilitating the restoration of epithelial homeostasis.

Our *in vitro* and *in vivo* experiments demonstrate the complex and seemingly contradictory effects of acetylcholine. On the one hand, it acts as a protective agent by restoring the integrity of tight junctions compromised by *P. gingivalis* infection. This is consistent with the spatial transcriptomics finding of a general upregulation of tight junction proteins in the periodontitis-affected epithelium and highlights its potential in promoting tissue repair and maintaining mucosal defense (France and Turner, 2017). On the other hand, acetylcholine exacerbates inflammation by upregulating pro-inflammatory chemokines (CXCL1 and CXCL8) and inflammasome components (NLRP3 and ASC) (Plemmenos et al., 2021; Fu and Wu, 2023). The fact that the topical application of exogenous acetylcholine ultimately aggravated periodontal destruction in a mouse model confirms that its pro-inflammatory effects outweigh its barrier-protective role in the context of active disease, leading to the heightened tissue damage and reduced alveolar bone density (Zhang et al., 2024).

Despite these compelling findings, our study has certain limitations. The causal relationships between specific cell types and neural signaling changes require further functional validation. Furthermore, our *in vitro* experiments utilized acetylcholine without the addition of AChE inhibitors. Given that keratinocytes may endogenously produce cholinesterases, the stability and effective concentration of acetylcholine in the culture medium could be impacted, which represents a limitation of this study. Additionally, although the mouse model is valuable, it may not fully recapitulate the complexity of human periodontal disease. Future studies should aim to elucidate the specific molecular pathways by which CHRNB1 and CHRNA5 mediate their pro-inflammatory and barrier-protective effects. Investigating whether pharmacological targeting of these receptors could be a viable therapeutic strategy for modulating periodontal inflammation and promoting tissue regeneration is a promising direction for future research.

## Data Availability

The RNA-seq data are listed under GSE297378 (GEO database), and the scRNA-seq data of gingiva are listed under GSE152042, 164241, 171213 and 207502 (GEO database). The spatial transcriptomics data are listed under HRA003217 in the Genome Sequence Archive (GSA) database.

## References

[B1] Alen N. V. (2022). The cholinergic anti-inflammatory pathway in humans: state-of-the-art review and future directions. Neurosci. Biobehav Rev. 136, 104622. 10.1016/j.neubiorev.2022.104622 35300992

[B2] Apatzidou D. A. Iskas A. Konstantinidis A. Alghamdi A. M. Tumelty M. Lappin D. F. (2018). Clinical associations between acetylcholine levels and cholinesterase activity in saliva and gingival crevicular fluid and periodontal diseases. J. Clin. Periodontol. 45 (10), 1173–1183. 10.1111/jcpe.12989 30022504

[B3] Arredondo J. Hall L. L. Ndoye A. Chernyavsky A. I. Jolkovsky D. L. Grando S. A. (2003). Muscarinic acetylcholine receptors regulating cell cycle progression are expressed in human gingival keratinocytes. J. Periodontal Res. 38 (1), 79–89. 10.1034/j.1600-0765.2003.01006.x 12558941

[B4] Barford K. Yap C. C. Dwyer N. D. Winckler B. (2017). The related neuronal endosomal proteins NEEP21 (Nsg1) and P19 (Nsg2) have divergent expression profiles *in vivo* . J. Comp. Neurol. 525 (8), 1861–1878. 10.1002/cne.24168 28299779 PMC5663458

[B5] Barreiro O. Martin P. Gonzalez-Amaro R. Sanchez-Madrid F. (2010). Molecular cues guiding inflammatory responses. Cardiovasc Res. 86 (2), 174–182. 10.1093/cvr/cvq001 20053659

[B6] Burke S. M. Avstrikova M. Noviello C. M. Mukhtasimova N. Changeux J. P. Thakur G. A. (2024). Structural mechanisms of alpha7 nicotinic receptor allosteric modulation and activation. Cell 187 (5), 1160–1176 e1121. 10.1016/j.cell.2024.01.032 38382524 PMC10950261

[B7] Caetano A. J. Yianni V. Volponi A. Booth V. D'Agostino E. M. Sharpe P. (2021). Defining human mesenchymal and epithelial heterogeneity in response to oral inflammatory disease. Elife 10, e62810. 10.7554/eLife.62810 33393902 PMC7781605

[B8] Chang A. M. Kantrong N. Darveau R. P. (2021). Maintaining homeostatic control of periodontal epithelial tissue. Periodontol 86 (1), 188–200. 10.1111/prd.12369 33690934

[B9] Chen Y. Wang H. Yang Q. Zhao W. Chen Y. Ni Q. (2022). Single-cell RNA landscape of the osteoimmunology microenvironment in periodontitis. Theranostics 12 (3), 1074–1096. 10.7150/thno.65694 35154475 PMC8771561

[B10] Chen Y. Fang Z. M. Yi X. Wei X. Jiang D. S. (2023). The interaction between ferroptosis and inflammatory signaling pathways. Cell Death Dis. 14 (3), 205. 10.1038/s41419-023-05716-0 36944609 PMC10030804

[B11] de Juan A. Ince L. M. Pick R. Chen C. S. Molica F. Zuchtriegel G. (2019). Artery-associated sympathetic innervation drives rhythmic vascular inflammation of arteries and veins. Circulation 140 (13), 1100–1114. 10.1161/CIRCULATIONAHA.119.040232 31401849 PMC6756975

[B12] Easter Q. T. Fernandes Matuck B. Beldorati Stark G. Worth C. L. Predeus A. V. Fremin B. (2024). Single-cell and spatially resolved interactomics of tooth-associated keratinocytes in periodontitis. Nat. Commun. 15 (1), 5016. 10.1038/s41467-024-49037-y 38876998 PMC11178863

[B13] Emanuel E. Arifuzzaman M. Artis D. (2024). Epithelial-neuronal-immune cell interactions: implications for immunity, inflammation, and tissue homeostasis at mucosal sites. J. Allergy Clin. Immunol. 153 (5), 1169–1180. 10.1016/j.jaci.2024.02.004 38369030 PMC11070312

[B14] Fernandez-Lainez C. Aan de Stegge M. Silva-Lagos L. A. Lopez-Velazquez G. de Vos P. (2023). β(2 → 1)-β(2 → 6) and β(2 → 1) fructans protect from impairment of intestinal tight junction's gene expression and attenuate human dendritic cell responses in a fructan-dependent fashion. Carbohydr. Polym. 320, 121259. 10.1016/j.carbpol.2023.121259 37659831

[B15] Ferreira-Vieira T. H. Guimaraes I. M. Silva F. R. Ribeiro F. M. (2016). Alzheimer's disease: targeting the cholinergic system. Curr. Neuropharmacol. 14 (1), 101–115. 10.2174/1570159x13666150716165726 26813123 PMC4787279

[B16] Forrester E. A. Benitez-Angeles M. Redford K. E. Rosenbaum T. Abbott G. W. Barrese V. (2024). Crucial role for sensory nerves and Na/H exchanger inhibition in dapagliflozin- and empagliflozin-induced arterial relaxation. Cardiovasc Res. 120 (14), 1811–1824. 10.1093/cvr/cvae156 39056245 PMC11587556

[B17] France M. M. Turner J. R. (2017). The mucosal barrier at a glance. J. Cell Sci. 130 (2), 307–314. 10.1242/jcs.193482 28062847 PMC5278669

[B18] Fu J. Wu H. (2023). Structural mechanisms of NLRP3 inflammasome assembly and activation. Annu. Rev. Immunol. 41, 301–316. 10.1146/annurev-immunol-081022-021207 36750315 PMC10159982

[B19] Guzman-Mejia F. Lopez-Rubalcava C. Gonzalez-Espinosa C. (2018). Stimulation of nAchRα7 receptor inhibits TNF synthesis and secretion in response to LPS treatment of mast cells by targeting ERK1/2 and TACE activation. J. Neuroimmune Pharmacol. 13 (1), 39–52. 10.1007/s11481-017-9760-7 28822039

[B20] Hodo T. W. de Aquino M. T. P. Shimamoto A. Shanker A. (2020). Critical neurotransmitters in the neuroimmune network. Front. Immunol. 11, 1869. 10.3389/fimmu.2020.01869 32973771 PMC7472989

[B21] Hone A. J. McIntosh J. M. (2023). Nicotinic acetylcholine receptors: therapeutic targets for novel ligands to treat pain and inflammation. Pharmacol. Res. 190, 106715. 10.1016/j.phrs.2023.106715 36868367 PMC10691827

[B22] Hulsbusch N. Solis G. P. Katanaev V. L. Stuermer C. A. (2015). Reggie-1/Flotillin-2 regulates integrin trafficking and focal adhesion turnover via Rab11a. Eur. J. Cell Biol. 94 (11), 531–545. 10.1016/j.ejcb.2015.07.003 26299802

[B23] Ilic K. Mlinac-Jerkovic K. Sedmak G. Rosenzweig I. Kalanj-Bognar S. (2021). Neuroplastin in human cognition: review of literature and future perspectives. Transl. Psychiatry 11 (1), 394. 10.1038/s41398-021-01509-1 34282131 PMC8289873

[B24] Kim T. S. Ikeuchi T. Theofilou V. I. Williams D. W. Greenwell-Wild T. June A. (2024). Epithelial-derived interleukin-23 promotes oral mucosal immunopathology. Immunity 57 (4), 859–875.e11. 10.1016/j.immuni.2024.02.020 38513665 PMC11058479

[B25] Korabecny J. Soukup O. (2021). Cholinesterase research. Biomolecules 11 (8), 1121. 10.3390/biom11081121 34439787 PMC8394027

[B26] Kosumi H. Watanabe M. Shinkuma S. Nohara T. Fujimura Y. Tsukiyama T. (2022). Wnt/beta-catenin signaling stabilizes hemidesmosomes in keratinocytes. J. Invest Dermatol 142 (6), 1576–1586 e1572. 10.1016/j.jid.2021.10.018 34742703

[B27] Lamont R. J. Fitzsimonds Z. R. Wang H. Gao S. (2022). Role of Porphyromonas gingivalis in oral and orodigestive squamous cell carcinoma. Periodontol. 2000 89 (1), 154–165. 10.1111/prd.12425 35244980 PMC9439709

[B28] Li W. Zhu H. Zou X. Ye H. Zhong J. Xiang S. (2025a). A brain-to-lung signal from GABAergic neurons to ADRB2(+) interstitial macrophages promotes pulmonary inflammatory responses. Immunity 58 (8), 2069–2085.e9. 10.1016/j.immuni.2025.05.005 40466637

[B29] Li Y. He X. Luo G. Zhao J. Bai G. Xu D. (2025b). Innovative strategies targeting oral microbial dysbiosis: unraveling mechanisms and advancing therapies for periodontitis. Front. Cell Infect. Microbiol. 15, 1556688. 10.3389/fcimb.2025.1556688 40370404 PMC12075390

[B30] Liccardo F. Luini A. Di Martino R. (2022). Endomembrane-based signaling by GPCRs and G-Proteins. Cells 11 (3), 528. 10.3390/cells11030528 35159337 PMC8834376

[B31] Mak P. Chang C. Pursell B. Mercurio A. M. (2013). Estrogen receptor beta sustains epithelial differentiation by regulating prolyl hydroxylase 2 transcription. Proc. Natl. Acad. Sci. U. S. A. 110 (12), 4708–4713. 10.1073/pnas.1221654110 23487784 PMC3607009

[B32] Meric P. Buduneli N. Kanmaz B. Gurlek O. Comlekoglu E. Calvert G. (2019). Cholinergic signalling mechanisms and early implant healing phases in healthy versus generalized aggressive periodontitis patients: a prospective, case-control study. J. Clin. Periodontol. 46 (11), 1155–1163. 10.1111/jcpe.13185 31444906

[B33] Moayedi Y. Michlig S. Park M. Koch A. Lumpkin E. A. (2022). Localization of TRP channels in healthy oral mucosa from human donors. eNeuro 9 (6), ENEURO.0328–21.2022. 10.1523/ENEURO.0328-21.2022 36635242 PMC9797210

[B34] Mulder K. Patel A. A. Kong W. T. Piot C. Halitzki E. Dunsmore G. (2021). Cross-tissue single-cell landscape of human monocytes and macrophages in health and disease. Immunity 54 (8), 1883–1900.e5. 10.1016/j.immuni.2021.07.007 34331874

[B35] Petsakou A. Liu Y. Liu Y. Comjean A. Hu Y. Perrimon N. (2023). Cholinergic neurons trigger epithelial Ca(2+) currents to heal the gut. Nature 623 (7985), 122–131. 10.1038/s41586-023-06627-y 37722602 PMC10699467

[B36] Plemmenos G. Evangeliou E. Polizogopoulos N. Chalazias A. Deligianni M. Piperi C. (2021). Central regulatory role of cytokines in periodontitis and targeting options. Curr. Med. Chem. 28 (15), 3032–3058. 10.2174/0929867327666200824112732 32838709

[B37] Sakallioglu E. E. Lutfioglu M. Sakallioglu U. Diraman E. Pamuk F. Odyakmaz S. (2008). Local peptidergic innervation of gingiva in smoking and non-smoking periodontitis patients. J. Periodontol. 79 (8), 1451–1456. 10.1902/jop.2008.070667 18672995

[B38] Sharma B. R. Kanneganti T. D. (2021). NLRP3 inflammasome in cancer and metabolic diseases. Nat. Immunol. 22 (5), 550–559. 10.1038/s41590-021-00886-5 33707781 PMC8132572

[B39] Shen Z. Zhang R. Huang Y. Chen J. Yu M. Li C. (2024). The spatial transcriptomic landscape of human gingiva in health and periodontitis. Sci. China Life Sci. 67 (4), 720–732. 10.1007/s11427-023-2467-1 38172357

[B40] Slots J. (2017). Periodontitis: facts, fallacies and the future. Periodontol 75 (1), 7–23. 10.1111/prd.12221 28758294

[B41] Takahashi N. Sulijaya B. Yamada-Hara M. Tsuzuno T. Tabeta K. Yamazaki K. (2019). Gingival epithelial barrier: regulation by beneficial and harmful microbes. Tissue Barriers 7 (3), e1651158. 10.1080/21688370.2019.1651158 31389292 PMC6748373

[B42] Walczak-Nowicka L. J. Herbet M. (2021). Acetylcholinesterase inhibitors in the treatment of neurodegenerative diseases and the role of acetylcholinesterase in their pathogenesis. Int. J. Mol. Sci. 22 (17), 9290. 10.3390/ijms22179290 34502198 PMC8430571

[B43] Wang H. Yu M. Ochani M. Amella C. A. Tanovic M. Susarla S. (2003). Nicotinic acetylcholine receptor alpha7 subunit is an essential regulator of inflammation. Nature 421 (6921), 384–388. 10.1038/nature01339 12508119

[B44] Wang S. Y. Cai Y. Hu X. Li F. Qian X. H. Xia L. Y. (2024). P. gingivalis in oral-prostate axis exacerbates benign prostatic hyperplasia via IL-6/IL-6R pathway. Mil. Med. Res. 11 (1), 30. 10.1186/s40779-024-00533-8 38764065 PMC11103868

[B45] Wen G. Eder K. Ringseis R. (2024). Comparative evaluation of the modulatory role of 1,25-dihydroxy-vitamin D(3) on endoplasmic reticulum stress-induced effects in 2D and 3D cultures of the intestinal porcine epithelial cell line IPEC-J2. J. Anim. Sci. Biotechnol. 15 (1), 153. 10.1186/s40104-024-01112-6 39521992 PMC11550553

[B46] Wessler I. Reinheimer T. Kilbinger H. Bittinger F. Kirkpatrick C. J. Saloga J. (2003). Increased acetylcholine levels in skin biopsies of patients with atopic dermatitis. Life Sci. 72 (18-19), 2169–2172. 10.1016/s0024-3205(03)00079-1 12628475

[B47] Williams D. W. Greenwell-Wild T. Brenchley L. Dutzan N. Overmiller A. Sawaya A. P. (2021). Human oral mucosa cell atlas reveals a stromal-neutrophil axis regulating tissue immunity. Cell 184 (15), 4090–4104.e15. 10.1016/j.cell.2021.05.013 34129837 PMC8359928

[B48] Xu S. Jiang C. Liu H. Zhang H. Liao H. Wang X. (2020). Integrin-α9 and its corresponding ligands play regulatory roles in chronic periodontitis. Inflammation 43 (4), 1488–1497. 10.1007/s10753-020-01226-9 32232710

[B49] Zhang M. Liu Y. Afzali H. Graves D. T. (2024). An update on periodontal inflammation and bone loss. Front. Immunol. 15, 1385436. 10.3389/fimmu.2024.1385436 38919613 PMC11196616

[B50] Zhu W. Huang X. (2023). Mural cell composition and functional analysis in the healing process of human gingiva from periodontal intrabony defects. Arch. Oral Biol. 150, 105687. 10.1016/j.archoralbio.2023.105687 36947913

